# Quantitative FDG PET Assessment for Oncology Therapy

**DOI:** 10.3390/cancers13040869

**Published:** 2021-02-19

**Authors:** Kenji Hirata, Nagara Tamaki

**Affiliations:** 1 Department of Diagnostic Imaging, Graduate School of Medicine, Hokkaido University, Sapporo 060-8638, Japan; khirata@med.hokudai.ac.jp; 2 Department of Radiology, Graduate School of Medical Science, Kyoto Prefectural University of Medicine, Kyoto 602-8566, Japan

**Keywords:** positron emission tomography (PET), ^18^F-FDG, tumour metabolism, radiomics

## Abstract

**Simple Summary:**

PET enables quantitative assessment of tumour biology in vivo. Accumulation of F-18 fluorodeoxyglucose (FDG) may reflect tumour metabolic activity. Quantitative assessment of FDG uptake can be applied for treatment monitoring. Numerous studies indicated biochemical change assessed by FDG-PET as a more sensitive marker than morphological change. Those with complete metabolic response after therapy may show better prognosis. Assessment of metabolic change may be performed using absolute FDG uptake or metabolic tumour volume. More recently, radiomics approaches have been applied to FDG PET. Texture analysis quantifies intratumoral heterogeneity in a voxel-by-voxel basis. Combined with various machine learning techniques, these new quantitative parameters hold a promise for assessing tissue characterization and predicting treatment effect, and could also be used for future prognosis of various tumours.

**Abstract:**

Positron emission tomography (PET) has unique characteristics for quantitative assessment of tumour biology in vivo. Accumulation of F-18 fluorodeoxyglucose (FDG) may reflect tumour characteristics based on its metabolic activity. Quantitative assessment of FDG uptake can often be applied for treatment monitoring after chemotherapy or chemoradiotherapy. Numerous studies indicated biochemical change assessed by FDG PET as a more sensitive marker than morphological change estimated by CT or MRI. In addition, those with complete metabolic response after therapy may show better disease-free survival and overall survival than those with other responses. Assessment of metabolic change may be performed using absolute FDG uptake in the tumour (standardized uptake value: SUV). In addition, volumetric parameters such as metabolic tumour volume (MTV) have been introduced for quantitative assessment of FDG uptake in tumour. More recently, radiomics approaches that focus on image-based precision medicine have been applied to FDG PET, as well as other radiological imaging. Among these, texture analysis extracts intratumoral heterogeneity on a voxel-by-voxel basis. Combined with various machine learning techniques, these new quantitative parameters hold a promise for assessing tissue characterization and predicting treatment effect, and could also be used for future prognosis of various tumours, although multicentre clinical trials are needed before application in clinical settings.

## 1. Introduction

Positron emission tomography (PET) is a clinical tool that can measure the distribution of radioactivity concentration in a space and has high capability for quantitation [1]. PET with radiolabelled glucose analogue (^18^F-fluorodeoxyglucose [FDG]) enables the visualization of metabolic rate of glucose in vivo [2]. FDG PET is different from CT, which reflects anatomical structure, and MRI, which mainly reflects anatomical structure and diffusion. PET can provide images of molecular and biological function in vivo [3]. FDG PET is widely used for tumour detection, initial staging, evaluation of treatment response, detection of recurrence, and prediction of patient outcome [4,5,6,7,8].

Quantitative PET imaging can be applied to several areas of oncology therapy [9]. Firstly, FDG PET images reflect genetic and/or histological status of the tumour to decide treatment strategy [10]. Secondly, it can be used for more accurate delineation of target volumes in radiotherapy planning, since quantitative images often provide a good tumour-to-background contrast [11]. Thirdly, quantitative imaging may be used for response monitoring and treatment stratification, by deciding the optimal treatment modality and the optimal dose [12,13,14]. Fourthly, quantitative imaging could be used for dose painting, with the radiation dose spatially redistributed throughout the target volume depending on the quantitative parameter maps [15]. Finally, quantitative assessment of characteristics may be used for accurate assessment of treatment effect, including complete response, partial response, stable disease, and tumour progression [16]. Appropriate treatment monitoring is valuable for next step treatment planning.

## 2. Basic Concepts for Quantitative FDG PET Assessment

### 2.1. Qualitative Vs. Quantitative Assessment

In general, PET images are visually interpreted by well-trained nuclear medicine physicians. Tumour is comprehensively diagnosed by taking into account clinical history, family history, symptoms, laboratory data, and other imaging examinations [17]. This process is called visual assessment or qualitative assessment.

The advantage of PET is its capability for quantitative assessment [18]. PET images are not only a picture but also data that reflect spatial distribution of radioactivity concentration [19]. This additional quantitative information would be beneficial in various clinical settings.

Qualitative and quantitative assessments each have their strengths and weaknesses. Qualitative assessment enables comprehensive decisions by medical knowledge and the doctor’s experiences, but it is subjective and less reproducible. In addition, clinical training is required. In contrast, quantitative assessment is objective and produces numeric indices that are easy to understand, even by non-specialists. However, the same lesion may be measured as different values due to various methods in the process of calculating the numerical values. As both are imperfect, qualitative and quantitative assessments should complement each other.

The following manuscript will focus on the characterization of each quantitative assessment of PET.

### 2.2. Type of Quantitative Assessment

In this section, we would like to discuss several techniques for the quantitative evaluation of PET in depth (Table 1). The most rigorous quantification is absolute (direct) measurement of glucose metabolism rate, which has the unit of mol/100 g tissue/min [20]. Absolute quantification of glucose metabolism can be performed using dynamic FDG PET scanning and a suitable kinetic model. However, such quantitation appears challenging and is not practical for the clinical setting, particularly in oncological areas [21,22,23]. This technique requires catheterization of an artery (usually, radial artery) during the PET examination for continuous arterial blood sampling, and dynamic scanning from the injection up to 60 minutes. Long dynamic scanning reduces hospital throughput. Since the field of view (FOV) of general PET scanners is about 15–20 centimetres in the body axis direction, most of the body cannot be covered during the dynamic scanning and thus can be only scanned as static acquisition. Therefore, the highly invasive, labour-intensive, and time-consuming characteristics of this technique is not applicable in oncology areas. This is the major reason why there is little accumulated clinical evidence regarding absolute quantification.

The recently emerged technique total-body PET has extremely long FOVs in the axial direction, in which half or the whole body is covered [24]. The widespread adoption of these scanner would enable a reassessment of the role of absolute quantification using PET.

In contrast, semiquantitative approaches have been widely used as a standard procedure in clinical practice.

The simplest quantification is a lesion-to-normal (L/N) ratio. This requires an interpreter to define a region of interest (ROI) in the lesion and reference tissue (e.g., muscle, liver, lung, blood pool). L/N ratio is the radioactivity concentration of the lesion divided by that of the reference tissue. The ROI definition process is usually performed manually. This method is rarely used with some exceptions. One of the reasons is that inter-observer variations are introduced depending on the reference ROI, as normal tissues such as muscle and liver which have, to some extent, heterogeneous activity. Exceptions are brain tumours, where L/N ratio calculated by the contralateral cerebral cortex or cerebellum is preferred to standardized uptake value (SUV) [25], and cardiac sarcoidosis, for which L/N ratio is calculated using the blood pool of the descending aorta or liver [26].

SUV is a more objective measurement that is more objective than L/N ratio. It represents radioactivity concentration in the lesion at a single time point. When the injected tracer is homogeneously distributed in the entire body, SUV is defined as 1. SUV is calculated using the following formula: SUV = [tissue tracer activity concentration [Bq/mL]]/([injected dose [Bq]]/[patient body weight [g]]) [27]. Calculating SUV does not require reference region of interest (ROI). SUVmax represents maximal SUV value in the lesion, which is independent of ROI size. Generally, SUVmax is most commonly used in clinical practice, since this is mostly simple, reproducible and readily available using commonly used software [8]. However, SUVmax is sensitive to image, noise and motion. In addition, its value is dependent on image quality by the PET-CT system. Note that state-of-art PET-CT scanners with high spatial resolution usually produce images with high SUVmax, and that direct comparison between different scanners may be impossible.

Peak SUV (SUVpeak) has been introduced to overcome the shortcomings of SUVmax, as a hybrid value of measuring the mean value of radiotracer uptake within and ROI surrounding the highest-intensity voxel (generally 1-cm^3^ ROI surrounding the voxel with the highest activity). SUVpeak has the features of being less susceptible to noise and scanner differences in spatial resolution. In addition, an index called SUL has also been proposed [8], standing for standardized uptake value by lean body mass, but is not widespread yet.

A common caveat for the SUV family is that it is affected by blood glucose [28] and uptake time (i.e., the time interval between injection and scanning) [27]. Methods have been proposed for calculating SUV by normalizing with blood glucose, especially for brain tumours [29,30], however, they raise another problem of possible introduction of SUV error under blood glucose measurement.

### 2.3. Volumetric Indices

The L/N ratio and SUVmax metrics indicate the concentration of a very small region within the tumour, and thus, do not take into account tumour volume. In this point, PET is different from CT or MRI, where size is usually measured by a major axis. Indices estimating tumour size into account have also been established for PET as well. Among the volumetric parameters of PET, metabolic tumour volume (MTV) and total lesion glycolysis (TLG) are most popular. MTV represents volume of the tumour with active FDG uptake (usually above a certain threshold, such as SUVmax ≥ 2.5 or SUVmax ≥ 40%). TLG is calculated by multiplying the SUVmean of the total tumour by its MTV. In other words, TLG is summed SUV within the lesion. MTV and TLG have been shown to correlate with risk in various tumours, such as non-small cell lung cancer [31,32,33], head and neck cancer [34], and soft tissue sarcoma [35].

A recent study has reported evidence of the ability of volumetric indices to predict prognosis [36]. Although the volumetric indices are very useful, they are rarely mentioned in daily radiology reports because there is no consensus on how to determine the tumour boundary. Another reason is that it cannot be measured as easily as SUVmax. Dedicated software is needed to measure MTV and TLG [37]. Even if using such software, time-consuming human interaction is often needed. For example, when a tumour and a physiological accumulation (bladder, brain, etc.) are very close to each other, the two masses have to be separated manually by specialists, which also reduces inter-operator reproducibility.

### 2.4. Radiomics

Radiomics is a recently emerged field [38,39], in which sequential processes are applied as a mathematical model to extract numerical values, so-called features, from radiological images, followed by machine learning to obtain clinically useful information. Using radiomics, it is possible to obtain numerical data regarding whether a lesion is spherical or rod-shaped, and whether the internal metabolism is homogeneous or heterogeneous. As the radiomic features are provided as numerical values, they can be easily understood by physicians as if the results of laboratory tests are interpreted. Radiomics was first introduced for use in CT/MRI/ultrasonography (US), and the concept was later applied to PET. To the best of our knowledge, radiomics was first used in PET in 2009 [40].

**Figure 1:**
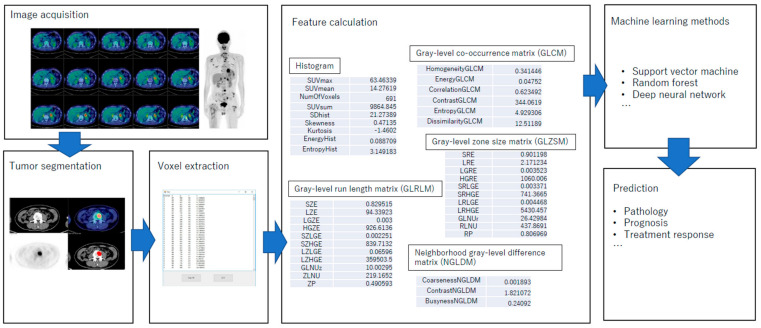
Radiomics showing how to perform tumour segmentation, voxel extraction from actual FDG PET images in order to assess feature calculation with various parameters using machine learning methods. These radiomic parameters will be applied for predictions of pathology, prognosis, and treatment response.

Figure 1 illustrates how radiomics is performed for tumour segmentation and voxel extraction from FDG PET images, feature calculation using various parameters and machine learning methods. These radiomic parameters will be applied for predictions of pathology, prognosis, and treatment response. Malignant tumours tend to have genetic heterogeneity [41] and to develop small hypoxic regions within the tumour [42,43,44,45,46], which may result in heterogeneous metabolism. It is reasonable to assume that the heterogeneity is associated with the aggressiveness of a tumour.

Attempts have been made to quantify intra-tumoural heterogeneity in a process termed texture analysis [47,48,49]. The simplest example may be to enclose the tumour with ROI, create a histogram of voxel values within the ROI, and calculate mean, standard deviation, energy, entropy, kurtosis, skewness, and so on. A cumulative histogram of SUV and its area under the curve, abbreviated as AUC-CSH, is also commonly used for this purpose [50].

In radiomics, a large number of indicators are calculated [51,52,53,54,55,56,57,58]. Previously, when only SUVmax was utilized, univariate analysis and receiver operator curve analysis were enough to determine whether the particular indicator was a useful predictor for histological or genetic characteristics and prognosis [59]. Current radiomics analysis produces a lot of variables. Care should be taken when multiple variables are analyzed. Overfitting easily occurs when the number of samples is small. When using machine learning techniques, the entire dataset must be split into the training and test datasets. According to TRIPOD guideline, generalization performance should be tested, if possible, using external datasets [60]. Principal component analysis and Lasso regression are useful methods for selecting significant variables.

There have been a lot of reports that have demonstrated usefulness of radiomics for clinical decision making. Even when focusing on lung cancer, PET radiomics are useful for predicting histology [61], prognosis [62], and tumour hypoxia [63].

However, despite ever-increasing evidence of its benefits, a lack of inter-scanner and inter-centre standardization has prevented radiomics from being incorporated into routine clinical practice. Radiomics indicators can be affected by tumour size, ROI size, image matrix and reconstruction parameters [64]. Researchers are making efforts to overcome the problems. Some examples are image acquisition guidelines by EANM [65], and post-reconstruction radiomics analysis guidelines of IBSI [66].

## 3. Clinical Applications of FDG PET

### 3.1. Tumour Characterization

FDG PET/CT is an important imaging method widely used for the functional metabolic and anatomical/morphological imaging of various types of malignant tumours and metastatic lesions. ^18^F-FDG PET/CT provides not only intuitive imaging differences through image comparisons, but also several metabolic parameters to distinguish metabolically active or inactive tumour tissues. FDG PET/CT is commonly used in the outcome study in various types of cancers. A number of reports revealed that MTV, as a surrogate for tumour cell number, has a strong prognostic value in diffuse large B-cell lymphoma [67,68]. Recently, this prognostic value was confirmed in a large patient population [69].

PET/CT has been widely used in clinical practice for the establishment of diagnosis, staging, treatment monitoring, and prognostic evaluation of non-small cell lung cancer (NSCLC). Several studies have confirmed that the FDG uptake of primary tumours is an independent risk factor for patients with early NSCLC [70,71], but its prognostic evaluation of NSCLC remains controversial [72]. Recent trial of radiomic signature based on PET/CT can be potentially used as a biomarker for risk stratification of the OS in patients with NSCLC [73].

FDG PET/CT has been used for identifying distant metastases in patients with locally advanced breast cancer. FDG PET/CT has been shown to offer more accurately staging, better tumour response prediction compared to anatomical imaging, and may have a role in radiation therapy planning [74,75,76,77]. A more recent study of inflammatory breast cancer indicated that higher tumour grade has higher SUVmax in involved regional lymph nodes and tended to have higher SUVmax in primary tumour. In addition, higher baseline SUVmax was associated with decreased OS in the advanced stage [78].

### 3.2. Optimal Assessment of Treatment Effect and Outcome

The World Health Organization (WHO) proposed the standardized criteria for assessing tumour response have been refined and simplified by the Response Evaluation Criteria in Solid Tumors (RECIST) guidelines, which were developed jointly by the European Organization for Research and Treatment of Cancer (EORTC), the National Cancer Institute (NCI) of the USA and the National Cancer Institute of Canada Clinical Trials group [79]. RECIST 1.0 criteria were initially published in 2000 and updated (RECIST 1.1) in 2009 [80]. Overall tumour burden is quantified by summing the size of lesions in a baseline scan before the start of a new therapy, and also response is then quantified by measuring the relative change of this sum of lesion sizes [79,80].

Assessment of treatment response with FDG PET-CT plays an important role for optimizing next treatment strategy and predicting patient outcome. Qualitative evaluation remains most commonly used approach in clinical practice by using visual comparison of the target lesion to the background, the mediastinal blood pool or the liver (Lugano Classification). PET response criteria have been established and modified by International Working Group Criteria (IWC) for evaluation of lymphoma treatment response [7,81]. The Deauville criteria are used to guide therapy during mid-treatment and end-treatment response. PET results are defined negative, when the residual lesion shows no FDG uptake (Deauville 1) or faint FDG uptake less than mediastinal blood pool (Deauville 2). PET results are defined positive when the residual lesion shows moderately higher (Deauville 4) or much higher (Deauville 5) than that of liver. Residual lesion with FDG uptake between the level of mediastinal blood pool and liver (Deauville 3) is regarded as positive when considering de-escalation of therapy or as negative when considering escalation of therapy. Similar approaches have been used for the quantitative assessment of FDG uptake with treatment monitoring for most malignancies when clinical values of such quantitative PET analysis for treatment response is shown in these areas.

Since PET has a unique character for quantitative assessment of tracer uptake as described before, a number of quantitative assessments of changes in FDG uptake have been applied for treatment monitoring. EORTC recommendations represented quantitative approach for response assessment with PET. Their recommendations used the percentage change in SUV under standard patient preparation [9] (Table 2).

Another set of quantitative criteria for response assessment with FDG PET is the Response Criteria in Solid Tumors (PERCIST) [8] (Table 2). They used lean body mass-normalized SUV (SUL) preferable to standard total body weight normalized SUV. The round-work of PERCIST may show practical advances for cancer care and research in many oncology areas.

Quantitative imaging using FDG PET, hypoxic PET, and MRI plays a major role in radiotherapy, where tissue sensitivity is related to microscopic processes that include metabolism, hypoxia, perfusion, and diffusivity. Quantitative imaging biomarkers (QIBs) can be used for response monitoring and treatment stratification, by choosing suitable treatment modality and optimal dose to the lesions [12,13,14,82].

Previous reports have indicated the prognostic value of quantitative assessment FDG PET. A systematic review of literature indicated prognostic effectiveness of FDG PET/CT parameters as biomarkers of overall survival, disease-free survival, and distant metastasis among patients with head and neck squamous cell cancer treated with surgery. Volumetric parameters (MTV and TLG) have been confirmed as relevant for identifying patients with a higher risk of postsurgical disease progression who could receive early therapeutic intervention to improve their prognosis [83]. Similar meta-analysis was reported and indicated prognostic value of quantitative FDG uptake parameters in non-small cell lung cancer [84] and uterine cervical cancer [85].

Diffuse large B-cell lymphoma (DLBCL) and Hodgkin lymphoma have been most extensively studied for risk stratification and outcome using FDG PET. Interim PET performed after 2 or 4 cycles of chemotherapy has been proposed as a tool for tailoring therapy. High-risk patients are not accurately identified by the current prognostic scoring systems [7]. Qualitative PET analysis using Deauville criteria as previously described has been used for accurate treatment effects and outcome analysis [7,86]. The prognostic role of quantitative PET parameters, in particular the metabolic volume (MTV), has been demonstrated in many lymphoma subtypes [87,88,89], including DLBCL [68,69,90,91]. MTV reflects the total volume of ^18^F-FDG–avid regions, and therefore, provides a comprehensive burden evaluation. Patients with a high burden are at higher risk for treatment failure and shorter survival than those with a low burden.

## 4. Conclusions

FDG PET can provide useful clinical information regarding tumour metabolism and aggressiveness in various types of cancers. In addition to qualitative assessment, PET images can be analysed by calculating numeral indices, including volumetric parameters in the tumours. Among the radiomics approaches focusing on image-based precision medicine, texture analysis can extract metabolic heterogeneity in a voxel-by-voxel basis. Combined with various machine learning techniques, these new quantitative parameters hold a promise for assessing tissue characterization, predicting treatment effect, and evaluating the prognosis of various tumours. Multi-centre clinical trials are needed before applied in the clinical settings.

## Figures and Tables

**Table 1:** Comparison among various quantitative measurements in ^18^F-fluorodeoxyglucose (FDG) positron emission tomography (PET).

Category	Example	Clinical Evidence	Pros	Cons
Direct quantification of glucose metabolism	Metabolic rate of glucose (mol /100 g tissue/min)	Little	Biologically understandable measurements	Invasive (arterial blood sampling), long scanning time, limiting number of patients
Semi-quantitative measurements	Lesion-to-normal (L/N) ratio	Medium	Easy to apply Used for brain tumor / cardiac sarcoidosis	Less reproducible than SUV due to reference ROI definition
Semi-quantitative measurements	Standardized uptake value (SUV), especially SUVmax	Largest	High inter-operator reproducibility Available in common software	Sensitive to noise, scanner resolution, and uptake time
Volumetric indices	Metabolic tumour volume (MTV) Total lesion glycolysis (TLG)	Large	Less sensitive to noise than SUVmax Combining tumour size and functional activity	Less reproducible than SUVmax No consensus on how to delineate tumour boundary Affected by nearby physiological uptake
Radiomics	Shape indices (e.g., sphericity) Textural features (e.g., entropy) Deep radiomics	Increasing	Extracting 100% information of the image Potential to classify lesions that are not distinguishable by human eye	Methodology has not been standardized (on-going).

**Table 2:** FDG criteria for response (modified from References [7,8]).

Response Classification	EORTC 1999	PERCIST 2009
PMD Progressive metabolic disease	Increase in SUV of greater than 25% - Or- Increase of the longest diameter by 20% - Or- new FDG avid lesion(s)	SUL increase by at least 30% and increase in by at least 0.8 SUL units of the target lesion - Or- Development of at least one new lesion - Or- Increase in target lesion size by 30% - Or- Unequivocal progression of nontarget lesions
SMD Stable metabolic disease	Increase of SUV by < 25% or decrease less than 15% - And- no increase in longest diameter > 20%	Increase or decrease of SUL by less than 30%
PMR Partial metabolic response	Decrease of SUV by 15–25% after one cycle of chemotherapy and > 25% after more than one treatment cycle	Decrease of SUL by ≥ 30% and at least 0.8 SUL units difference - And- No new FDG-avid lesions, - And- No increase in size > 30% of the target lesion - And- No increase in SUL or size of non-target lesion
CMR Complete metabolic response	Resolution of FDG uptake (indistinguishable from surrounding normal tissue)	FDG uptake indistinguishable from surrounding background - And- SUL less than liver

## Data Availability

Not applicable.
